# Professional helpers' experiences of assisting the bereaved after drug-related deaths: A knowledge gap

**DOI:** 10.1177/14550725221085345

**Published:** 2022-05-02

**Authors:** Monika Alvestad Reime, Hilde-Margit Løseth, Sari Kaarina Lindeman, Kristine Berg Titlestad, Kari Dyregrov, Lillian Bruland Selseng

**Affiliations:** 1657 Western Norway University of Applied Sciences, Bergen, Norway; 1657 Western Norway University of Applied Sciences, Bergen, Norway; 1657 Western Norway University of Applied Sciences, Bergen, Norway; 1657 Western Norway University of Applied Sciences, Bergen, Norway; 1657 Western Norway University of Applied Sciences, Bergen, Norway; 1657 Western Norway University of Applied Sciences, Bergen, Norway

**Keywords:** bereavement, drug-related death, help system, overdose, professionals, systematic review

## Abstract

**Background and aims:** Drug-related death (DRD) is a major public health concern in the Nordic countries, in the rest of Europe and in the US. After a DRD, approximately 10–15 next of kin will be left behind. People bereaved after sudden and unexpected deaths have a documented higher risk of reduced quality of life, daily functioning, and early death. It is important to know the resources professional helpers have available to them, the barriers and possibilities they face in their work, and how they can respond to the needs of the bereaved. This knowledge can help prevent severe health and social consequences of bereavement following a DRD. In this systematic review, the aim was to explore knowledge regarding professional helpers’ experiences of providing assistance to people bereaved after a DRD. **Methods:** Inclusion criteria were empirical studies of professional helpers’ first-person perspectives on meeting the bereaved after a DRD. Quantitative, qualitative, and mixed-method studies were included. **Results:** The results show that there are no studies addressing professional helpers’ experiences of providing assistance to the bereaved after a DRD. **Conclusion:** There is a vital need to develop more knowledge of professional helpers’ perspectives. This knowledge is important not only to improve education and the quality of health and social services, but also to help raise awareness of the bereaved after a DRD.

Drug-related death (DRD) poses a growing problem in Europe and in North America, and represents a significant public health challenge. In 2019, the mortality rate due to overdose were in the European Union estimated to be 14.8 deaths per millon population aged 15–64 years (European Monitoring Centre for Drugs and Drug Addiction (EMDCCA), 2021). In the US in 2019, the aged-adjusted rate for overdose deaths was 21.6% per 100,000 (Centers for Disease Control and Prevention (CDC), 2022a). The overdose mortality development in the US in the last decade has been described as reaching epidemic proportions. Recent data from the US indicate a substantial increase in DRDs before and during the COVID-19 pandemic, and the largest number of drug overdoses in a 12-month period ever recorded Centers for Disease Control and Prevention (CDC), (2022b). In addition to deaths caused by overdoses, drug abuse is also the cause of other unnatural deaths. This further increases the drug-related mortality rate (Robertson et al., 2019). Every year around 300 people die from drug-related causes in Norway. The other Scandinavian countries have comparable mortality rates (Dyregrov et al., 2020).

In this article, we define DRDs as deaths caused by the intake of substances classed as narcotics, and deaths when the cause of death is violence, accidents, infectious disease, and other health disorders which in different ways may be linked to drug use (Norwegian Directorate of Health, 2014, p. 14). After a DRD, it is estimated that there will be at least 10–15 next of kin, i.e., family members and close friends, left behind (Dyregrov et al., 2020). This systematic review explores what is known about professional helpers’ experiences of providing assistance to this group of bereaved people. By the term “professional helper” we refer to employees with a formal education (Abbott, 1988), working in different services that meet with the bereaved, for example local health and welfare services, mental health services, and first-response services.

Previous research has documented that after sudden and unexpected deaths (e.g., murder, suicide, sudden infant death, unnatural deaths), there is an increased risk of physical and mental illnesses (Dyregrov et al., 2003; Stroebe et al., 2017) and lower levels of health-related quality of life (Song et al., 2010). After unnatural deaths, those bereaved seem to experience more grief symptoms such as general health problems, post-traumatic distress, and complicated grief reactions (Dyregrov et al., 2015; Dyregrov et al., 2003; Titlestad, Lindeman, et al., 2021). There is also an increased risk of prolonged grief disorder (PGD), which is the most common form of complicated grief (Djelantik et al., 2020). PGD is characterised by persistent separation distress combined with cognitive, emotional, and behavioural symptoms, resulting in severe functional impairment for at least six months post loss (World Health Organization (WHO), 2021). The results from a recent study of bereaved parents after a DRD show a significant high risk for prolonged grief symptoms (Titlestad, Schmid, et al., 2021). Also, a recent registry study from Norway that encompasses all Norwegian parents with at least one child above the age of 15 years in the period 1986–2015 showed that parents who had lost a child to DRD had a higher mortality rate than both non-bereaved parents and parents who had lost a child to other modes of death. The mortality was particularly high in the first two years after a loss (Christiansen et al., 2020).

The dual process model of coping with bereavement is a scientific model that describes how complicated grief reactions can be reduced by balancing loss-orienting and re-orienting coping, i.e., thoughts and actions (Stroebe & Schut, 1999, 2010). Inspired by previous knowledge from drug-related and bereavement research, Dyregrov has developed a theoretical model of drug-death bereavement, i.e., the “special grief”. This model integrates the strains of the demanding situation before death, with various grief reactions post loss. The strains are related to helping the drug user while still alive and to the mental and societal challenges faced, such as several years of uncertainty, despair, stigma, hopelessness, and anticipatory grief (Dyregrov et al., 2020). Anticipatory grief is related to the anticipation and fear of death and is often described in studies of persons with cognitive decline or cancer and in studies of the bereaved after a DRD (Templeton et al., 2017; Titlestad, Mellingen, et al., 2021). The consequences of the “special grief” after a DRD imply that the strain experienced before and after the death may contribute to emotional overload (Stroebe & Schut, 2016) and increase the risk of developing a prolonged grief disorder. Knowledge about grief processes and the different kinds of strains that can follow the “special grief” directs attention to the professional helpers’ role, responsibility, and attitudes towards bereaved persons after a DRD. The existing knowledge about DRD actualises that severe health implications can follow and addresses the need for targeted help for those left behind (Christiansen et al., 2020).

The limited research on DRD bereavement indicates a lack of assistance from the helping system. The only existing systematic review on DRD points to an emotional and existential overload in the bereaved, but lacks understanding and help from the support systems (Titlestad, Lindeman, et al., 2021). Titlestad, Lindeman, et al. (2021), among others, document the discrepancy between help wished for by the bereaved after unnatural deaths and the help that they received. To some extent, the bereaved connect the lack of help and understanding to stigmatising attitudes from professional helpers (Dyregrov, 2002; Titlestad, Lindeman, et al., 2021). In relation to this, research has reported that stigmatisation is a barrier both to seeking help and to receiving help in situations where the cause of death is related to potential or perceived stigmatised behaviour such as suicide or drug use (Hanschmidt et al., 2016; Scocco et al., 2019; Titlestad, Lindeman, et al., 2021).

In a large study in England and Scotland, Valentine et al. (2018) documented several shortcomings in the support systems’ response to persons bereaved by a drug-related and/or alcohol-related death of someone close. The study was based on data from semi-structured interviews with bereaved people (*n* = 106) and six focus group interviews with practitioners from different support services such as the police, clergy, health service, bereavement service, funeral service, and alcohol and drug treatment services. Results showed that the practitioners had a poor understanding of what kind of help the bereaved person needed and lacked knowledge of how support services could be involved. This might hamper the collaboration between different support services and hinder the offer and access to adequate services. The study also reported that the bereaved themselves felt a lack of understanding from the support system and experienced fragmented, discriminatory, and inadequate services.

Peter Cartwright (2020) discusses how to support people bereaved by substance-related deaths (drug- and/or alcohol-related deaths). Based on semi-structured interviews with 20 bereaved persons after a substance-related death, Cartwright outlines three useful theoretical perspectives for bereavement support: the dual process model (Stroebe & Schut, 1999, 2010), the theory of continuing bonds (Klass et al., 2014), and attachment theory (Bowlby, 1969). Cartwright also outlines concrete measures to understand and manage substance-related bereavement from the practitioner's perspective. Particularly, he points to the importance of practitioners being aware of the special characteristics of substance-related bereavement, and the need of specialist knowledge and referrals to adequate services (Cartwright, 2019, 2020). Cartwright (2015) developed guidelines for practitioners whose work (either paid or voluntary) brings them into contact with the bereaved after substance-related deaths. The guidelines recommend that practitioners be kind and compassionate, consider thoroughly what kind of language they use and the attitudes they communicate, and be aware that the bereaved are unique people with different needs and reactions, that they should have a responsibility to contribute and finally to be familiar with relevant services and work together with other practitioners (Cartwright, 2015).

Yule and Levin (2019) consider providers’ experiences after a drug-overdose death. The focus is primarily on the emotional reactions and management strategies after experiencing a patient’s death, but the authors also point to the importance of interaction with the bereaved family. According to Yule and Levin, to date no studies have addressed this topic. They therefore draw on insights from the existing suicide literature, which they argue has similarities with drug-overdose death particularly relating to the sudden and unexpected nature of the deaths together with the moral and societal stigma associated with the cause of death.

Although research from related fields can inform our knowledge on bereavement after DRDs, there is a need to explore systematically the extent of available research on professional helpers’ experiences of encountering the bereaved after a DRD. Such studies are essential to inform further research and point out clinical implications.

## Aims of the review

To respond adequately to the needs of the bereaved after a DRD and to develop services that are supportive and responsive to the needs of the bereaved, it is necessary to understand the experience of professional helpers encountering the bereaved after a DRD, to know what resources they have available, and what they perceive as challenges and possibilities in their work. A systematic review will contribute to collecting, appraising, and synthesising all available studies on the topic. By following a strict and reproducible methodological framework, it will be a reliable source for further discussions, the development of new research questions, and the improvement and innovation of targeted welfare services (Lund et al., 2016).

This review aims to identify qualitative studies, quantitative studies and mixed-method studies that shed light on the professional helpers’ experiences of providing assistance to the bereaved after a DRD, and eventually to synthesise these studies.

## Method

The Preferred Reporting Items for Systematic reviews and Meta-Analyses (PRISMA) statement guided the review (Liberati et al., 2009). The review protocol was registered in the PROSPERO International prospective register of systematic reviews in August 2020 (Registration number: CRD42020191572).

### Search strategy

Nine electronic databases were searched: MEDLINE, Scopus, Embase, PsycINFO, CINAHL, SocINDEX, ASSIA (Applied Social Sciences Index and Abstracts), ProQuest (ProQuest Dissertations and Thesis Global) and ORIA (a Norwegian database for books, scientific articles, and grey literature). The search strategy (see Supplement I online) was carefully developed by searching for keywords in similar articles, and through discussions in the research group, with librarians and with peers at conferences. The search strategy was piloted and adjusted accordingly. The search terms were developed to identify quantitative, qualitative, and mixed-method research exploring experiences of providing assistance to the bereaved after a DRD from a professional helper's perspective. Final search terms were adapted to fit the search engines of different databases. Search terms were combined with Boolean operators in the following way: “drug-related” OR “drug abuse*” OR “drug addict*” OR “drug depend*” OR “drug misuse*” OR “drug usage” OR “drug use disorder” OR “illicit drug*” OR “substance-related disorders” OR “substance abuse*” OR “substance addict*” OR “substance depend*” OR “substance use” OR “alcoholism” OR “alcohol abuse*” OR “alcohol addict*” OR “alcohol depend*” OR “alcohol misuse*” OR “alcohol-related disorder*” OR “alcohol use disorder*” AND “death*” OR “overdose*” OR “deceased” AND “bereave*” OR “grief*” OR “grieving” OR “mourning*” OR “loss”. Also, Norwegian search terms were developed based on the English search terms to fit the database for Norwegian literature (ORIA).

The searches were conducted by two trained academic librarians, commencing on 24 August and finishing on 9 October 2020. In addition, we searched for relevant citations in the full-text articles that we screened, in a review relevant to the topic (Titlestad, Lindeman, et al., 2021), and we carried out a citation search in Google Scholar for the same articles. We also carried out manual searches in the following journals for the past five years: *Drugs: Education, Prevention & Policy*, *Journal of Substance Use*, *OMEGA – Journal of Death and Dying and Death Studies*. Study alerts for new studies relevant to our research question were enabled. No limitations were imposed due to publication date.

### Inclusion/exclusion criteria

To address the aims and objectives of the review, the included studies were required to meet the following inclusion criteria:

- The study is an empirical study.
- The sample for the study is professional helpers who relate to the bereaved after a DRD in their work. By professional helpers, we refer to: “exclusive occupational groups applying somewhat abstract knowledge to particular cases” (Abbott, 1988, p. 8).
- The outcome of the study is based on the first-person perspective of professional helpers regarding their experiences with providing assistance.
- The design of the study is qualitative, quantitative, or mixed-methods.
- The study is published in peer-reviewed articles, books, or dissertations.
- The study is in English or Norwegian.

Studies were excluded if:

- Professional helpers’ experiences with assistance after a DRD are impossible to distinguish from their experiences with assistance after other types of substance-related bereavement (such as alcohol).
- The study is not peer-reviewed (such as a master's thesis or reports).
- The study is of low methodological quality.
- The study has a high risk of bias.

### Search outcome and study selection

**Figure 1.**
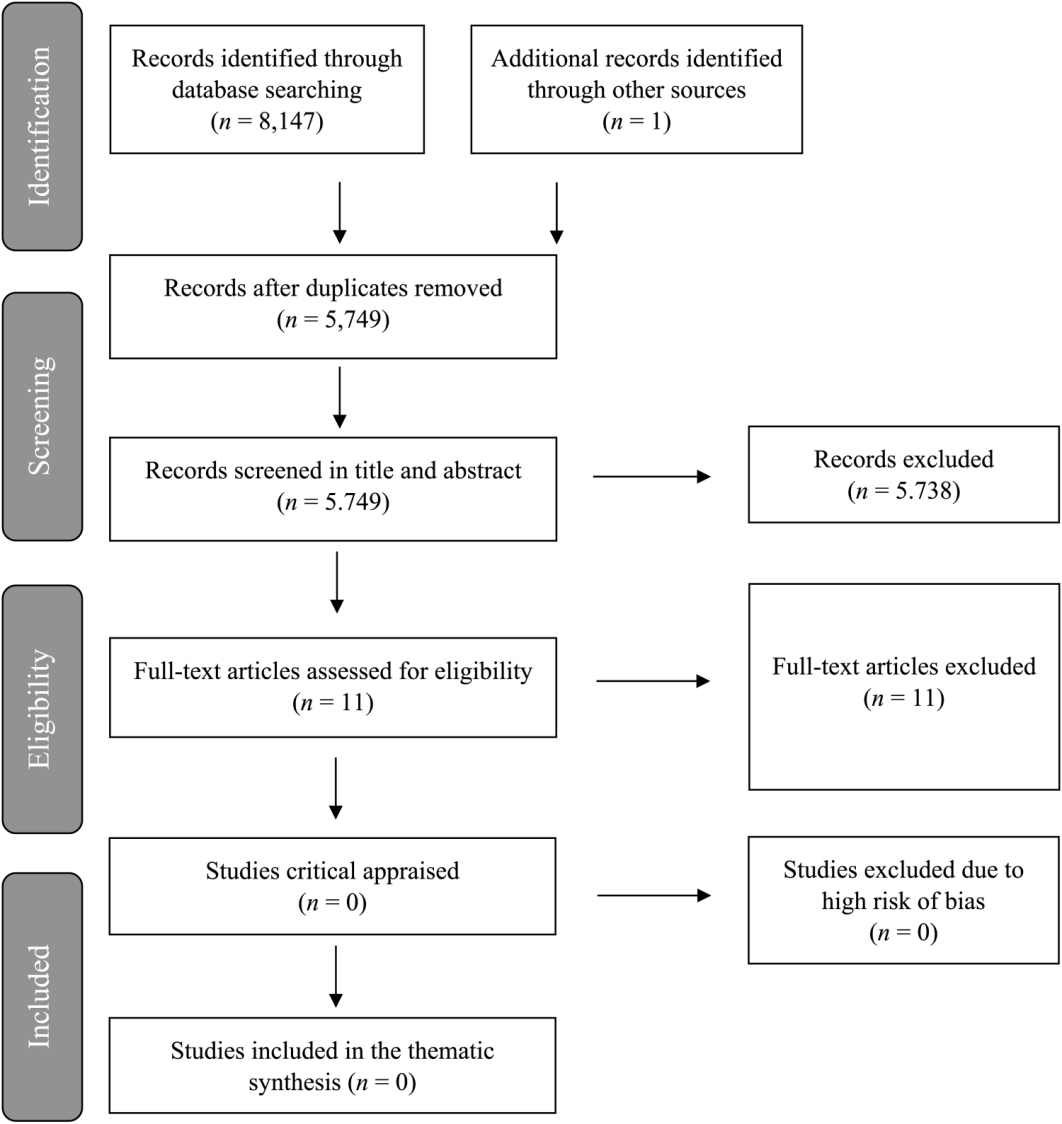
The Prisma flow diagram.

References from the electronic searches (8,147) and manual searches (1) were exported into a reference citation manager (Rayyan). This gave a total of 8,148 studies exported to Rayyan. When duplicates were removed 5,749 studies were left for screening. Two commissions with two researchers in each (MAR & SKL and LBS & HML) independently examined the titles and the abstracts of all references against the inclusion/exclusion criteria. Any disagreements were resolved through discussion until consensus was reached. After screening the titles and abstracts, 5,738 studies were excluded, and 11 studies were left for full-text review. Two commissions with two researchers in each (MAR & LBS and HML & SKL) independently carried out the full-text review, and any disagreements were resolved through discussion. No studies fulfilled the inclusion criteria. The main reason for exclusion at this stage was due to the study's sample or type of study (e.g., theoretical article). All full-text articles excluded at this stage of the selection process are presented in an “Excluded Studies” table together with the reason for the exclusion (see Supplement II in the online material). Finally, no studies were left for critical appraisal. A PRISMA flow diagram has been developed for documenting the review process (see Figure 1).

## Results

The results of this systematic review show that there are no studies addressing professional helpers’ experiences of providing assistance to the bereaved after a DRD.

## Discussion

### Knowledge needs and clinical implications

The lack of knowledge of the professional helper's perspective documented in this systematic review represents a serious challenge to the quality of health and social care, and to the overall aim of developing targeted and supportive welfare services. Given the high proportion of DRDs in Europe (European Monitoring Centre for Drugs and Drug Addiction (EMDCCA), 2021) and the extremely challenging situation in the US (Centers for Disease Control and Prevention (CDC), 2022b), there is an urgent need for professional helpers in health and social services to be aware of all the people left behind, and the severe challenges they face in the aftermath of death and the potentially harmful health implications.

After sudden and unexpected deaths, the bereaved have an increased risk of long-term serious grief- and trauma-related reactions and reduced quality of life and daily functioning (Dyregrov et al., 2015; Dyregrov et al., 2003; Titlestad, Lindeman, et al., 2021; Titlestad, Schmid, et al., 2021). Such deaths are also associated with prolonged grief disorder (PGD) (Djelantik et al., 2020), post-traumatic stress disorder (PTSD) and depression (Li et al., 2003), as well as increased mortality (Christiansen et al., 2020). In addition, research also documents that after sudden and unexpected deaths the bereaved often fail to receive the help they need if the support system does not have a proactive approach to those in need of help (Dyregrov, Berntsen, & Silviken, 2014; Dyregrov & Dyregrov, 2008; Dyregrov, Kristensen, et al., 2014).

The limited research on the bereaved after a DRD, indicates that this group of people experience significant burdens in relation to emotional and existential loads, a lack of help from support systems, and stigmatisation (Templeton et al., 2016; Titlestad, Lindeman, et al., 2021; Titlestad, Mellingen et al., 2021; Valentine et al., 2018). To respond adequately to the needs of the bereaved after a DRD and to develop health and social services in the community that are supportive and responsive, there is a need for knowledge on how the professional helpers experience encountering the bereaved after a DRD, what resources they have available and what they perceive as challenging and as possible in their work. This systematic review shows that there is an urgent need to develop research that addresses these questions. This knowledge is important to improve and develop services and educational systems, to inform policy- and decision-makers, and to help increase the awareness of a potentially marginalised and stigmatised group of bereaved people at risk of severe health implications and even early death.

### Why does drug-related bereavement need exclusive attention?

In the choice of the review question and the eligibility criteria, we decided to focus exclusively on drug-death bereavement, meaning that we excluded studies that combined experiences of drug- and alcohol-related death. We are aware that many people with problematic drug use often use a mixture of different substances also including alcohol, hence professional helpers will sometimes work simultaneously with people bereaved through both drug- and alcohol-related death. Also, many people die from alcohol-related causes each year. In the year 2020, 386 deaths were registered as alcohol-related deaths in Norway (Norwegian Institute of Public Health, 2022). Knowledge from professional helpers’ experiences of providing assistance to the bereaved after alcohol-related death can inform practice and can to a given extent be transferable to work with people bereaved through DRD, but there are some important differences that we will argue make it important to gain specific knowledge on professional helpers’ experiences with DRD bereavement.

Particularly we will point to the association between DRD and criminality. One of the barriers to access the help system for the bereaved after a DRD that was identified in studies of the experiences of the bereaved is stigma, both self-imposed and from the professional helpers (Dyregrov et al., 2020; Dyregrov & Selseng, 2021; Templeton et al., 2016; Titlestad, Mellingen, et al., 2021; Valentine et al., 2018). Self-imposed stigma is identified among the bereaved and is related to feelings of shame and guilt, particularly because drug use is a criminal activity and the drug user is often perceived to be a threat to society (Guy & Holloway, 2007; Templeton et al., 2017). After the death, the belief that DRD is a self-inflicted action associated with deviance and morally unacceptable behaviour contributes to making the grief less legitimate and thereby less “grieveable” for the bereaved (Guy & Holloway, 2007).

Research that points to addiction stigma being worsened by criminalisation (Corrigan et al., 2017) has informed our choice to direct our attention to DRD bereavement. The war on drugs by the Nixon administration in the 1970s gave a boost to the understanding of drug use as a criminal activity and has continued to have a significant effect on the treatment of drug users in the US and the attitudes towards drug users in society as a whole. Despite reforms this century that aim to reduce penalties and find alternatives to incarceration (Corrigan et al., 2017), research from the US, the UK, and Norway still shows that people who use illegal drugs are stigmatised (Dyregrov et al., 2020; Dyregrov & Selseng, 2021; Feigelman et al., 2011; Templeton et al., 2016; Titlestad, Mellingen, et al., 2021; Valentine et al., 2018).

Based on interviews with 32 adults in England and Scotland who were bereaved after a fatal overdose, Templeton et al. (2017) argue that bereavement after an overdose is different from bereavement after deaths related to other substances, such as alcohol. They point to circumstances surrounding a fatal overdose which often leads to a range of questions for the bereaved. For example, if others were involved or responsible for the death, or how the body of the deceased is handled by the policy or the coroners. Furthermore, Templeton et al. (2017) point to stigma and complex emotions following this cause of death, such as guilt, self-blame, and asking whether they could have done anything different when the deceased was still alive. Titlestad, Mellingen, et al. (2021), who explore the experiences of DRD-bereaved parents, find that self-imposed stigma is also related to feelings of guilt and shame for having failed as a parent, and being unable to protect their child from the drug use, and subsequent death.

Several studies report that those bereaved after a DRD experience stigma from the professional helpers both before and after the death (Templeton et al., 2016; Titlestad, Mellingen, et al., 2021; Valentine et al., 2018). For example, one study reported that the bereaved felt that professional helpers focussed more on the fact that the deceased was a drug addict than recognising their grief and helping them with the grieving process (Templeton et al., 2016). Each cause of death has a different status, and DRDs have a low status (Guy & Holloway, 2007). This can affect how the help system responds to the bereaved. For example, stigma can reduce access to services and healthcare because the health services themselves avoid stigmatised groups (Sheehan & Corrigan, 2020). The process by which family members are “forced” to share the discredit that is associated with their stigmatised family member is highlighted by Goffman (1963) and is also relevant for professional helpers encountering stigmatised groups. Sheehan and Corrigan (2020) utilise the concept of associative stigma (Halter, 2008) when they address how family members, friends, and health workers are tarnished by stigma through their connection with the stigmatised individual.

Societal and associative stigma together with self-imposed stigma can be a barrier to seeking and receiving help, as well as a challenge to the quality of services for the bereaved. The association between DRD, criminality and unacceptable behaviour worsens the stigma and the challenges faced by the bereaved after a DRD and makes addressing how professional helpers relate to this in their daily practice an urgent matter.

### Quality of the review and limitations

One of the strengths of systematic reviews is the strict methodological framework following from the PRISMA approach (Liberati et al., 2009). All steps in the approach are followed and documented, making the study transparent, reproduceable and reliable. In addition, the electronic database searches were conducted by trained academic librarians. This is recommended to ensure the quality of searches (Malterud, 2019). Before the final search, a pilot study was carried out to ensure that the search string and the search terms were adequate for the aim of the article. We discussed the results of the pilot both in the project research group and with experts in an international conference in the autumn of 2019, and we adjusted the search terms and search string accordingly. Experiences from the pilot and the adjustments contribute to strengthen the overall quality of the review.

Despite the strict methodological framework, piloting, and adjustment process, there could still be studies that we have overlooked. There may be search terms that we should have included in the search string, or databases that we should have searched. However, the choice of databases was well informed and was made in dialogue with the trained academic librarians. Together the nine electronic databases, manual searches and reference searches, are comprehensive and strengthen the methodological quality.

This review is limited to exploring professional helpers’ perspectives, which means that we excluded literature on peer support groups. We are aware that peer support is an important supplement to paid and formal professional work, but we believe that it is important to document professional helpers’ experiences. Professional helpers have a formal education, and they apply their theoretical and discretionary knowledge to particular cases (Abbott, 1998). Based on this formal knowledge, professional helpers are mandated to prevent and solve societal challenges. In this work, professional helpers control an enormous number of resources, and they make decisions that can have severe consequences for people's lives (Molander & Terum, 2008). Research on professional practices and experiences is therefore of importance when human challenges in society are addressed. This knowledge is particularly important when the phenomena under study (DRD) is described as an acute health challenge in society and calls for urgent attention by policy and practitioners (Centers for Disease Control and Prevention (CDC), 2022b). 

## Conclusion and implications for further research

This systematic review has shown that there is no research addressing professional helpers’ experiences of providing assistance to the bereaved after a DRD. Research has shown that the DRD-bereaved need help because they suffer severe health challenges and have an increased risk of premature death. Research has also revealed barriers for the bereaved in the health and welfare services. Therefore, there is a vital need for more knowledge about the professional helpers’ own experiences, their attitudes, knowledge, and practices when relating to the bereaved after a DRD. This knowledge is crucial to develop targeted and supportive services and for the development of educational systems and theoretical frameworks aimed at improving professional helpers’ formal knowledge of the needs and challenges of this group of bereaved people. Knowledge of the resources available to the professional helpers, the barriers and possibilities they face in their work, and how they are able to respond to the needs of the bereaved is important. In addition, knowledge about stigma and attitudes, both societal and associative, is important to reduce barriers to access to services and to improve quality of services.

Professional helpers are employed in different welfare systems and in different social and cultural contexts. Different contexts may affect attitudes and stigma towards drug use and DRD, the expectations of the responsibility and role of professional helpers, and the tasks and services provided by the professional helpers. We therefore recommend research across nations and welfare state regimes, eventually producing comparative research. Professional helpers work in different services and have different educational backgrounds. We recommend that further research takes this into consideration in the research design and takes a multidisciplinary approach to recruitment in order to gain as much information as possible about different experiences. Knowledge on interprofessional collaboration would be affordable to enhance knowledge on the different services involved and to facilitate the development of collaborative measures and organisational structures to improve collaboration and information among the professional helpers. Knowledge of professional helpers’ experiences of collaboration with peer support groups would be preferable, as this could strengthen the overall service to this group of bereaved people.

Knowledge of professional helpers’ experiences is important to improve education and the quality of community health and social services for the bereaved after a DRD. However, knowledge is also important to raise awareness and recognition of the bereaved after a DRD and to prevent the possible severe health and social consequences of the “special grief” following a DRD.

## Supplemental Material

sj-pdf-1-nad-10.1177_14550725221085345 - Supplemental material for Professional helpers' experiences of assisting the bereaved after drug-related deaths: A knowledge gap Click here for additional data file. Supplemental material, sj-pdf-1-nad-10.1177_14550725221085345 for Professional helpers' experiences of assisting the bereaved after drug-related deaths: A knowledge gap by Monika Alvestad Reime, Hilde-Margit Løseth, Sari Kaarina Lindeman, Kristine Berg Titlestad, Kari Dyregrov and Lillian Bruland Selseng in Nordic Studies on Alcohol and Drugs

sj-docx-2-nad-10.1177_14550725221085345 - Supplemental material for Professional helpers' experiences of assisting the bereaved after drug-related deaths: A knowledge gap Click here for additional data file. Supplemental material, sj-docx-2-nad-10.1177_14550725221085345 for Professional helpers' experiences of assisting the bereaved after drug-related deaths: A knowledge gap by Monika Alvestad Reime, Hilde-Margit Løseth, Sari Kaarina Lindeman, Kristine Berg Titlestad, Kari Dyregrov and Lillian Bruland Selseng in Nordic Studies on Alcohol and Drugs

## References

[bibr1-14550725221085345] Abbott A. (1988). The system of professions: An essay on the division of expert labor. University of Chicago Press.

[bibr2-14550725221085345] Bowlby J. (1969). Attachment and loss: Volume I: Attachment (pp. 1–401). The Hogarth Press and the Institute of Psycho-Analysis.

[bibr3-14550725221085345] Cartwright P. (2015). Bereaved through substance use: Guidelines for those whose work brings them into contact with adults bereaved after a drug or alcohol-related death. University of Bath.

[bibr4-14550725221085345] Cartwright P. (2019). How helpful is counselling for people bereaved through a substance-related death? Bereavement Care, 38(1), 23–32. 10.1080/02682621.2019.1587869

[bibr5-14550725221085345] Cartwright P. (2020). Supporting people bereaved through a drug- or alcohol-related death. Jessica Kingsley Publishers.

[bibr6-14550725221085345] Centers for Disease Control and Prevention (CDC). (2022a). https://www.cdc.gov/drugoverdose/deaths/index.html

[bibr6a-14550725221085345] Centers for Disease Control and Prevention (CDC). (2022b). *Overdose Deaths Accelerating During COVID-19*. https://www.cdc.gov/media/releases/2020/p1218-overdose-deaths-covid-19.html

[bibr7-14550725221085345] Christiansen S. G. Reneflot A. Stene-Larsen K. Johan Hauge L. (2020). Parental mortality following the loss of a child to a drug-related death. European Journal of Public Health, 30(6), 1098–1102. 10.1093/eurpub/ckaa094 32535625

[bibr8-14550725221085345] Corrigan P. Schomerus G. Smelson D. (2017). Are some of the stigmas of addictions culturally sanctioned? The British Journal of Psychiatry, 210(3), 180–181. 10.1192/bjp.bp.116.185421 28249945

[bibr9-14550725221085345] Djelantik A. M. J. Smid G. E. Mroz A. Kleber R. J. Boelen P. A. (2020). The prevalence of prolonged grief disorder in bereaved individuals following unnatural losses: Systematic review and meta regression analysis. Journal of Affective Disorders, 265, 146–156. 10.1016/j.jad.2020.01.034 32090736

[bibr10-14550725221085345] Dyregrov K. (2002). Assistance from local authorities versus survivors’ needs for support after suicide. Death Studies, 26(8), 647–669. 10.1080/07481180290088356 12243197

[bibr11-14550725221085345] Dyregrov K. Berntsen G. Silviken A. (2014). Needs and barriers for professional help: A qualitative study of bereaved in Sami areas. Suicidology Online, 5(1), 47–58.

[bibr12-14550725221085345] Dyregrov K. Dyregrov A. (2008). Effective grief and bereavement support: The role of family, friends, colleagues, schools and support professionals. Jessica Kingsley Publishers.

[bibr13-14550725221085345] Dyregrov K. Dyregrov A. Kristensen P. (2015). Traumatic bereavement and terror: The psychosocial impact on parents and siblings 1.5 years after the July 2011 terror killings in Norway. Journal of Loss and Trauma, 20(6), 556–576. 10.1080/15325024.2014.957603

[bibr14-14550725221085345] Dyregrov K. Kristensen P. Johnsen I. Dyregrov A. (2014). Hvordan fungerte den psyko-sosiale oppfølgingen for etterlatte etter 22. juli terroren? [How did the psychosocial follow up for the bereaved after the July 22 terror work?]. Scandinavian Psychologist, 1, Article e7. 10.15714/scandpsychol.1.e7

[bibr15-14550725221085345] Dyregrov K. Møgster B. Løseth H.-M. Lorås L. Titlestad K. B. (2020). The special grief following drug related deaths. Addiction Research & Theory, *28*(5), 1–10. 10.1080/16066359.2019.1679122

[bibr16-14550725221085345] Dyregrov K. Nordanger D. Dyregrov A. (2003). Predictors of psychosocial distress after suicide, SIDS and accidents. Death Studies, 27(2), 143–165. 10.1080/07481180302892 12678058

[bibr17-14550725221085345] Dyregrov K. Selseng L. B. (2021). “Nothing to mourn, He was just a drug addict”: Stigma towards people bereaved by drug-related death. Addiction Research & Theory, *30*(1), 1–11. 10.1080/16066359.2021.1912327

[bibr18-14550725221085345] European Monitoring Centre for Drugs and Drug Addiction (EMDCCA). (2021). *European Drug Report* . https://www.emcdda.europa.eu/system/files/publications/13838/TDAT21001ENN.pdf

[bibr19-14550725221085345] Feigelman W. Jordan J. R. Gorman B. S. (2011). Parental grief after a child’s drug death compared to other death. Causes: Investigating a greatly neglected bereavement population. OMEGA - Journal of Death and Dying, 63(4), 291–316. 10.2190/om.63.4.a 22010370

[bibr20-14550725221085345] Goffman E. (1963). Stigma: Notes on the management of spoiled identity. Prentice-Hall.

[bibr21-14550725221085345] Guy P. Holloway M. (2007). Drug-related deaths and the “special deaths” of late modernity. Sociology, 41(1), 83–96. 10.1177/0038038507074717

[bibr22-14550725221085345] Halter M. J. (2008). Perceived characteristics of psychiatric nurses: Stigma by association. Archives of Psychiatric Nursing, 22(1), 20–26. 10.1016/j.apnu.2007.03.003 18207053

[bibr23-14550725221085345] Hanschmidt F. Lehnig F. Riedel-Heller S. G. Kersting A. (2016). The stigma of suicide survivorship and related consequences: A systematic review. PLoS ONE, 11(9), Article e0162688. 10.1371/journal.pone.0162688 27657887 PMC5033475

[bibr24-14550725221085345] Klass D. Silverman P. R. Nickman S. (2014). Continuing bonds: New understandings of grief. Taylor & Francis.

[bibr25-14550725221085345] Li J. Precht D. H. Mortensen P. B. Olsen J. (2003). Mortality in parents after death of a child in Denmark: A nationwide follow-up study. The Lancet, 361(9355), 363–367. 10.1016/s0140-6736(03)12387-2 12573371

[bibr26-14550725221085345] Liberati A. Altman D. G. Tetzlaff J. Mulrow C. Gøtzsche P. C. Ioannidis J. P. , Clarke, M., Devereaux, P. J., Kleijnen, J., & Moher D. (2009). The PRISMA statement for reporting systematic reviews and meta-analyses of studies that evaluate health care interventions: Explanation and elaboration. Journal of Clinical Epidemiology, 62(10), e1–e34. 10.7326/0003-4819-151-4-200908180-00136 19631507

[bibr27-14550725221085345] Lund H. Juhl C. Christensen R. (2016). Systematic reviews and research waste. The Lancet, 387(10014), 123–124. 10.1016/s0140-6736(15)01354-9 26841992

[bibr28-14550725221085345] Malterud K. (2019). Qualitative metasynthesis: A research method for medicine and health sciences. Routledge. 10.4324/9780429026348

[bibr29-14550725221085345] Molander A. Terum L. (2008). Profesjonsstudier–en introduksjon [Studies of professions: An introduction]. In Molander A. Terum L. I. (Eds.), Profesjonsstudier [Studies of professions] (pp. 13–29). Universitetsforlaget.

[bibr30-14550725221085345] Norwegian Directorate of Health. (2014). Nasjonal overdose strategi 2014–2017 [National overdose strategy 2014–2017] . Norwegian Directorate of Health. https://www.regjeringen.no/contentassets/43121155483947d79316af20c68e6d7d/overdosestrategi_230414.pdf

[bibr31-14550725221085345] Norwegian Institute of Public Health (NIPH). (2022). *Statistics on causes of death 2020* . FHI. https://www.fhi.no/hn/helseregistre-og-registre/dodsarsaksregisteret/tall-fra-dodsarsaksregisteret-for-2020/

[bibr32-14550725221085345] Robertson R. Bird S. M. McAuley A. (2019). Drug-related deaths: A wider view is necessary. Addiction, 114(8), 1504–1504. 10.1111/add.14627 30955207

[bibr33-14550725221085345] Scocco P. Preti A. Totaro S. Corrigan P. Castriotta C. Team S. (2019). Stigma, grief and depressive symptoms in help-seeking people bereaved through suicide. Journal of Affective Disorders, 244, 223–230. 10.1016/j.jad.2018.10.098 30366261

[bibr34-14550725221085345] Sheehan L. Corrigan P. (2020). Stigma of disease and its impact on health. In C. S. Richards & L.M. Cohen (Eds) The Wiley Encyclopedia of Health Psychology (Vol. 3, pp. 57–65). Wiley. 10.1002/9781119057840.ch139

[bibr35-14550725221085345] Song J. Floyd F. J. Seltzer M. M. Greenberg J. S. Hong J. (2010). Long-term effects of child death on parents’ health-related quality of life: A dyadic analysis. Family Relations, 59(3), 269–282. 10.1111/j.1741-3729.2010.00601.x 20676393 PMC2910450

[bibr36-14550725221085345] Stroebe M. S. Schut H. (1999). The dual process model of coping with bereavement: Rationale and description. Death Studies, 23(3), 197–224. 10.1080/074811899201046 10848151

[bibr37-14550725221085345] Stroebe M. S. Schut H. (2010). The dual process model of coping with bereavement: A decade on. OMEGA-Journal of Death and Dying, 61(4), 273–289. 10.2190/om.61.4.a 21058610

[bibr38-14550725221085345] Stroebe M. S. Schut H. (2016). Overload: A missing link in the dual process model? OMEGA-Journal of Death and Dying, 74(1), 96–109. 10.1177/0030222816666540

[bibr39-14550725221085345] Stroebe M. S. Stroebe W. Schut H. Boerner K. (2017). Grief is not a disease but bereavement merits medical awareness. The Lancet, 389(10067), 347–349. 10.1016/s0140-6736(17)30189-7 28137681

[bibr40-14550725221085345] Templeton L. Ford A. McKell J. Valentine C. Walter T. Velleman R. , Bauld, L., Hay, G., & Hollywood J. (2016). Bereavement through substance use: Findings from an interview study with adults in England and Scotland. Addiction Research & Theory, 24(5), 341–354. 10.3109/16066359.2016.1153632

[bibr41-14550725221085345] Templeton L. Valentine C. McKell J. Ford A. Velleman R. Walter T. , Hay, G., Bauld, L., & Hollywood J. (2017). Bereavement following a fatal overdose: The experiences of adults in England and Scotland. Drugs: Education, Prevention and Policy, 24(1), 58–66. 10.3109/09687637.2015.1127328

[bibr42-14550725221085345] Titlestad K. B. Lindeman S. K. Lund H. Dyregrov K. (2021). How do family members experience drug death bereavement? A systematic review of the literature. Death Studies, *45*(7), 1–14. 10.1080/07481187.2019.1649085 31390307

[bibr43-14550725221085345] Titlestad K. B. Mellingen S. Stroebe M. Dyregrov K. (2021). Sounds of silence. The “special grief” of drug-death bereaved parents: A qualitative study. Addiction Research & Theory, *29*(2), 1–11. 10.1080/16066359.2020.1751827

[bibr44-14550725221085345] Titlestad K. B. Schmid M. T. Dyregrov K. (2021). Prevalence and predictors of prolonged grief symptoms among those bereaved from a drug-related death in a convenience sample of Norwegian parents: A cross-sectional study. Death Studies, Advanced online publication. 10.1080/07481187.2020.1867255 33427100

[bibr45-14550725221085345] Valentine C. McKell J. Ford A. (2018). Service failures and challenges in responding to people bereaved through drugs and alcohol: An interprofessional analysis. Journal of Interprofessional Care, 32(3), 295–303. 10.1080/13561820.2017.1415312 29257913

[bibr46-14550725221085345] World Health Organization (WHO). (2021). *International statistical classification of diseases and related health problems (ICD)* . WHO. https://www.who.int/standards/classifications/classification-of-diseases

[bibr47-14550725221085345] Yule A. M. Levin F. R. (2019). Supporting providers after drug overdose death. American Journal of Psychiatry, 176(3), 173–178. 10.1176/appi.ajp.2018.18070794 30818987 PMC6436101

