# Supplementary material for: Professional helpers' experiences of assisting the bereaved after drug-related deaths: A knowledge gap
Source: Nordisk Alkohol Nark. 2022 May 2;39(4):453–65. doi: 10.1177/14550725221085345 (PMC9379292; doi:10.1177/14550725221085345)
Supplement: sj-pdf-1-nad-10.1177_14550725221085345 - Supplemental material for Professional helpers' experiences of assisting the bereaved after drug-related deaths: A knowledge gap [file sj-pdf-1-nad-10.1177_14550725221085345.pdf]

**Databases**<br>**Search date**	**Search strategy**	**Number of hits**
**Medline**<br>2020-09-09	Database: Ovid MEDLINE(R) and Epub Ahead of Print, In-Process & Other Non-Indexed Citations and Daily <1946 to September 08, 2020> Search Strategy:<br>--<br>1 drug-related.mp. (47470)<br>2 exp Drug Abuse/ or drug abuse*.mp. (287689)<br>3 exp Drug Addiction/ or drug addict*.mp. (283750)<br>4 drug depend*.mp. (6531)<br>5 drug dependency.mp. or exp Drug Dependency/ (574)<br>6 drug misuse*.mp. (3649)<br>7 drug usage.mp. or exp Drug Usage/ (1795)<br>8 ("drug use disorder*" or "drug use").mp. (46039)<br>9 illicit drug*.mp. or exp Illicit Drugs/ (21085)<br>10 substance-related disorder*.mp. (96375)<br>11 substance abuse*.mp. (53763)<br>12 substance addict*.mp. (631)<br>13 substance depend*.mp. (3047)<br>14 exp "Substance Use Disorder"/ or "substance use disorder*".mp. (284950)<br>15 "substance use*".mp. (37921)<br>16 alcoholism.mp. or exp Alcoholism/ (84280)<br>17 exp Alcohol Abuse/ or alcohol abuse*.mp. (84638)<br>18 alcohol addict*.mp. (1645)<br>19 exp "Alcohol Use Disorder"/ (75189)<br>20 alcohol depend*.mp. (12771)<br>21 alcohol misuse*.mp. (2838)<br>22 alcohol-related disorder*.mp. (5734)<br>23 1 or 2 or 3 or 4 or 5 or 6 or 7 or 8 or 9 or 10 or 11 or 12 or 13 or 14 or 15 or 16 or 17 or 18 or 19 or 20 or 21 or 22 (411011)<br>24 bereav*.mp. or exp Bereavement/ (16577)	1,058

	25 exp Grief/ or grief*.mp. (12343)<br>26 grieving.mp. (1545)<br>27 mourning.mp. (1557)<br>28 loss.mp. (975839)<br>29 24 or 25 or 26 or 27 or 28 (990720)<br>30 death*.mp. or exp "Death and Dying"/ (920668)<br>31 exp Drug Overdoses/ or overdose*.mp. (25011)<br>32 deceased.mp. (20096)<br>33 30 or 31 or 32 (952577)<br>34 23 and 29 and 33 (1058)	
**Scopus**<br>2020-09-05	TITLE-ABS-KEY ("drug-related" OR "drug abuse*" OR "drug addict*" OR "drug depend*" OR "drug misuse*" OR "drug usage" OR "drug use disorder*" OR "illicit drug*" OR "substance-related disorders" OR "substance abuse*" OR "substance addict*" OR "substance depend*" OR "substance use" OR alcoholism OR "alcohol abuse*" OR "alcohol addict*" OR "alcohol depend*" OR "alcohol misuse*" OR "alcohol-related disorder*" OR "alcohol use disorder*") AND TITLE-ABS-KEY (bereave* OR grief* OR grieving OR mourning* OR loss) AND TITLE-ABS-KEY (death* OR overdose* OR deceased)	1,327

**Embase**<br>2020-09-10	Database: Embase <1974 to 2020 Week 36><br>Search Strategy:<br>--<br>1 drug-related.mp. (27860)<br>2 drug addict*.mp. (15294)<br>3 drug depend*.mp. (66637)<br>4 exp drug dependence/ (234459)<br>5 drug misuse.mp. or exp drug misuse/ (10365)<br>6 drug usage.mp. (2600)<br>7 exp drug abuse/ or drug abuse*.mp. (133917)<br>8 "drug use disorder*".mp. (1487)<br>9 exp illicit drug/ or illicit drug*.mp. (22875)<br>10 substance-related disorder*.mp. (4582)<br>11 exp substance abuse/ or substance abuse*.mp. (70100)<br>12 substance addict*.mp. or exp Substance Addiction/ (234890)<br>13 substance depend*.mp. (4309)<br>14 "substance use*".mp. or exp "substance use"/ (528591)<br>15 exp alcoholism/ or alcoholism.mp. (127369)<br>16 exp alcohol abuse/ or alcohol abuse*.mp. (51154)<br>17 alcohol addict*.mp. (2757)<br>18 "alcohol use disorder*".mp. (16176)<br>19 alcohol depend*.mp. (19621)<br>20 alcohol misuse*.mp. (3710)<br>21 alcohol-related disorder*.mp. (1338)<br>22 1 or 2 or 3 or 4 or 5 or 6 or 7 or 8 or 9 or 10 or 11 or 12 or 13 or 14 or 15 or 16 or 17 or 18 or 19 or 20 or 21 (863872)<br>23 exp bereavement/ or bereave*.mp. (13245)<br>24 grief*.mp. or exp grief/ (15496)<br>25 grieving.mp. (2001)<br>26 exp mourning/ or mourning.mp. (2767)	3,705

	27 loss.mp. (1308455)<br>28 23 or 24 or 25 or 26 or 27 (1327706)<br>29 exp death/ (722350)<br>30 death*.mp. (1378731)<br>31 exp drug overdose/ or overdose*.mp. (44376)<br>32 deceased.mp. (38939)<br>33 29 or 30 or 31 or 32 (1614034)<br>34 22 and 28 and 33 (3705)	
**PsycInfo**<br>2020-09-05	Database: APA PsycInfo <1806 to August Week 5 2020> Search Strategy:<br>--<br>1 drug-related.mp. (4126)<br>2 exp Drug Abuse/ or drug abuse*.mp. (59596)<br>3 exp Drug Addiction/ or drug addict*.mp. (23863)<br>4 drug depend*.mp. (15625)<br>5 drug dependency.mp. or exp Drug Dependency/ (13110)<br>6 drug misuse*.mp. (1621)<br>7 drug usage.mp. or exp Drug Usage/ (101430)<br>8 "drug use disorder*".mp. (1452)<br>9 illicit drug*.mp. (7827)<br>10 substance-related disorder*.mp. (37042)<br>11 substance abuse*.mp. (44024)<br>12 substance addict*.mp. (640)<br>13 substance depend*.mp. (3473)<br>14 exp "Substance Use Disorder"/ or "substance use disorder*".mp. (135220)<br>15 "substance use".mp. (60069)<br>16 alcoholism.mp. or exp Alcoholism/ (52256)<br>17 exp Alcohol Abuse/ or alcohol abuse*.mp. (53625)<br>18 alcohol addict*.mp. (1787)<br>19 exp "Alcohol Use Disorder"/ (52817)<br>20 alcohol depend*.mp. (12219)	576

	21 alcohol misuse*.mp. (2379)<br>22 alcohol-related disorder*.mp. (3353)<br>23 1 or 2 or 3 or 4 or 5 or 6 or 7 or 8 or 9 or 10 or 11 or 12 or 13 or 14 or 15 or 16 or 17 or 18 or 19 or 20 or 21 or 22 (261471)<br>24 bereave*.mp. or exp Bereavement/ (18730)<br>25 exp Grief/ or grief*.mp. (19325)<br>26 grieving.mp. (2977)<br>27 mourning.mp. (5022)<br>28 loss.mp. (116290)<br>29 24 or 25 or 26 or 27 or 28 (132443)<br>30 death*.mp. or exp "Death and Dying"/ (108220)<br>31 exp Drug Overdoses/ or overdose*.mp. (4908)<br>32 deceased.mp. (3959)<br>33 30 or 31 or 32 (113163)<br>34 23 and 29 and 33 (576)	
**Cinahl**<br>2020-09-09	(MH "Substance Use Disorders") OR (MH "Substance Abuse") OR (MH "Substance Dependence") OR (MH "Alcoholism") OR (MH "Alcohol Abuse") OR (MH "Alcohol-Related Disorders") OR ""drug-related"" OR ""drug abuse*"" OR ""drug addict*"" OR ""drug depend*"" OR ""drug misuse*"" OR ""drug usage"" OR ""drug use disorder*"" OR ""illicit drug*"" OR ""substance-related disorder*"" OR ""substance abuse*"" OR ""substance addict*"" OR ""substance depend*"" OR ""substance use*"" OR "alcoholism" OR ""alcohol abuse*"" OR ""alcohol addict*"" OR ""alcohol depend*"" OR ""alcohol misuse*"" OR ""alcohol-related disorder*"" OR ""alcohol use disorder*""<br>AND<br>MH "Bereavement" OR MH "Grief" OR MH "Complicated Grief" OR MH "Disenfranchised Grief" OR "bereave*" OR "grief*" OR "grieving" OR "mourning*" OR "loss"<br>AND<br>MH "Death" OR "death*" OR MH "Overdose" OR "overdose*" OR "deceased"	298
**SocIndex**<br>2020-08-24	DE "DRUG abuse" OR DE "SUBSTANCE abuse" OR DE "DRUG addiction" OR DE "ALCOHOLISM" OR DE "DRUG abusers" OR DE "DRUG addicts" OR DE "ADDICTS" OR DE "ADULT children of drug addicts" OR DE "CHILDREN of drug addicts" OR DE "PARENTS of drug addicts" OR DE "WOMEN drug addicts" OR DE "SUBSTANCE-induced disorders" OR DE "DRUG addicts -- Alcohol use" OR "drug-related" OR "drug abuse*" OR "drug addict*" OR "drug	298

	depend*" OR "drug misuse*" OR "drug usage" OR "drug use disorder*" OR "illicit drug*" OR "substance-related disorder*" OR "substance abuse*" OR "substance addict*" OR "substance depend*" OR "substance use*" OR alcoholism OR “alcohol abuse*” OR “alcohol addict*” OR “alcohol depend*” OR “alcohol misuse*” OR "alcohol-related disorder*" OR "alcohol use disorder*"<br>AND<br>DE "LOSS (Psychology)" OR DE "BEREAVEMENT" OR DE "GRIEF" OR bereave* OR grief* OR grieving OR mourning* OR loss<br>AND<br>DE "DEATH" OR DE "NARCOTICS -- Overdose" OR death* OR overdose* OR deceased	
**ASSIA from ProQuest**, 2020-09-10	((MAINSUBJECT.EXACT.EXPLODE("Drug related problems") OR noft(drug-related)) OR (noft("drug abuse*") OR (MAINSUBJECT.EXACT.EXPLODE("Drug abuse") OR MAINSUBJECT.EXACT.EXPLODE("Drug abusers"))) OR ((MAINSUBJECT.EXACT.EXPLODE("Drug addiction") OR MAINSUBJECT.EXACT.EXPLODE("Drug addicts")) OR noft("drug addict*")) OR (MAINSUBJECT.EXACT.EXPLODE("Drug dependency") OR noft("drug depend*")) OR noft("drug misuse*") OR noft("drug usage") OR noft("drug use disorder") OR noft("illicit drug*") OR noft("substance-related disorder*") OR ((MAINSUBJECT.EXACT.EXPLODE("Substance abuse disorders") OR MAINSUBJECT.EXACT.EXPLODE("Substance abusers") OR MAINSUBJECT.EXACT.EXPLODE("Substance abuse")) OR noft("substance abuse*")) OR noft("substance addict*") OR (MAINSUBJECT.EXACT.EXPLODE("Substance dependency") OR noft("substance depend*")) OR noft("substance use*") OR (MAINSUBJECT.EXACT.EXPLODE("Alcoholism") OR noft(alcoholism)) OR (MAINSUBJECT.EXACT.EXPLODE("Alcohol abuse") OR noft("alcohol abuse*")) OR noft("alcohol addict*") OR (MAINSUBJECT.EXACT.EXPLODE("Alcohol dependence") OR noft("Alcohol depend*")) OR noft("Alcohol misuse*") OR (MAINSUBJECT.EXACT.EXPLODE("Alcohol related disorders") OR noft("Alcohol-related disorder*")) OR noft("alcohol use disorder*")) AND (noft(deceased) OR (noft(death*) OR MAINSUBJECT.EXACT.EXPLODE("Death")) OR (noft(overdose*) OR MAINSUBJECT.EXACT.EXPLODE("Overdoses"))) AND ((MAINSUBJECT.EXACT.EXPLODE("Bereavement") OR noft(bereave*)) OR (MAINSUBJECT.EXACT.EXPLODE("Grief") OR noft(grief*)) OR noft(grieving) OR (MAINSUBJECT.EXACT.EXPLODE("Mourning") OR noft(mourning*)) OR (MAINSUBJECT.EXACT.EXPLODE("Loss") OR noft(loss)))	190
**ProQuest Dissertations &**	noft("drug-related" OR "drug abuse*" OR "drug addict*" OR "drug depend*" OR "drug misuse*" OR "drug usage" OR "drug use disorder*" OR "illicit drug*" OR "substance-related disorder*" OR "substance abuse*" OR "substance addict*" OR "substance depend*" OR "substance use*" OR alcoholism OR “alcohol abuse*” OR	112

**Thesis Global**<br>2020-09-09	“alcohol addict*” OR “alcohol depend*” OR “alcohol misuse*” OR "alcohol-related disorder*" OR "alcohol use disorder*")<br>AND<br>noft(bereave* OR grief* OR grieving OR mourning* OR loss) AND<br>noft(death* OR overdose* OR deceased)	
**ORIA**<br>2020-10-09	(rusmi* OR rusavh* OR stoffmisbruk OR stoffavh* OR narko* OR alkohol*) AND<br>(sorg OR tap OR sørgende OR etterlatt*) AND<br>(overdo* OR død OR dødsfall OR avdød OR avdøde OR daud)	583
