# Supplementary material for: Professional helpers' experiences of assisting the bereaved after drug-related deaths: A knowledge gap
Source: Nordisk Alkohol Nark. 2022 May 2;39(4):453–65. doi: 10.1177/14550725221085345 (PMC9379292; doi:10.1177/14550725221085345)
Supplement: sj-docx-2-nad-10.1177_14550725221085345 - Supplemental material for Professional helpers' experiences of assisting the bereaved after drug-related deaths: A knowledge gap [file sj-docx-2-nad-10.1177_14550725221085345.docx]

**Supplement II.** Excluded studies.

| **References** | **Exclusion due to** |
| --- | --- |
| Cartwright, P. (2020). *Supporting People Bereaved through a Drug-or Alcohol-Related Death.* Jessica Kingsley Publishers.    McAuley, A., & Forsyth, A. J. (2011). The impact of drug-related death on staff who have experienced it as part of their caseload: an exploratory study. *Journal of Substance Use*, *16*(1), 68-78.  McKell, J., Valentine, C., & Walter, T. (2017). Dealing with substance-related deaths. In *Families Bereaved by Alcohol or Drugs* (pp. 143-163). Routledge. | Empirical data from bereaved’ perspective, not professionals.  Focus on helpers as bereaved from DRD.  This is a book chapter. Does not distinguish between alcohol and drug related bereavement. |
| Valentine, C & Bauld, L (2017). Conclusion (pp.183-187). In *Families Bereaved by Alcohol or Drugs.* Routledge. | This is conclusion chapter in book (Valentine (ed), 2017), and not an empirical article. |
| Winship, G. (1993). Facing death in the therapeutic community. *Therapeutic Communities*.  Shear, M. K., Muldberg, S., & Periyakoil, V. (2017). Supporting patients who are bereaved. *BMJ*, *358*.  Schut, H., & Stroebe, M. (2011). Challenges in evaluating adult bereavement services. *Bereavement Care*, *30*(1), 5-9 | Focus on helpers as bereaved from DRD.  This is a theoretical article (on grief generally)  This is atheoretical article. |
|  |  |
| Urmanche, A. A. (2020). Bearing witness to the epidemic: Supporting clinicians after a client overdose death. *Practice Innovations*, *5*(4), 275.  Valentine, C., McKell, J., & Ford, A. (2018). Service failures and challenges in responding to people bereaved through drugs and alcohol: An interprofessional analysis. *Journal of interprofessional care, 32*(3), 295-303.  Valentine, C. (ed) (2017). *Families Bereaved by Alcohol or Drugs*. Routledge.  Yule, A. M., & Levin, F. R. (2019). Supporting providers after drug overdose death. *American Journal of Psychiatry, 176*(3), 173-178. https://doi.org/10.1176/appi.ajp.2018.18070794 | This is a theoretical article.  Does not distinguish between alcohol and drug related bereavement.  This is a book, relevant chapters are reviewed and covered by this list.  This is a theoretical article (with a vignette). |
|  |  |
|  |  |
|  |  |
|  |  |
|  |  |
|  |  |
|  |  |
